# Awareness of infection care terms among outpatients and carers in a public health facility: a cross-sectional survey

**DOI:** 10.12688/wellcomeopenres.20162.1

**Published:** 2023-12-14

**Authors:** Ebruphiyo Ruth Useh, Bongeka Mfeketo, Okuhle Mbengo, Innocent Karangwa, Timothy Pennel, Adam Boutall, Salome Maswime, Linda Pohl, Esmita Charani, Marc Mendelson, Oluchi Mbamalu

**Affiliations:** 1 School of Public Health, Faculty of Health Sciences, University of Cape Town, Rondebosch, Western Cape, 7925, South Africa; 2 Division of Infectious Diseases & HIV Medicine, Department of Medicine, Groote Schuur Hospital, University of Cape Town, Rondebosch, Western Cape, 7925, South Africa; 3 AFDA Film School, Cape Town, Western Cape, 7925, South Africa; 4 Statistical Consulting Unit, Department of Statistical Sciences, University of Cape Town, Rondebosch, Western Cape, 7700, South Africa; 5 Chris Barnard Division of Cardiothoracic Surgery, Groote Schuur Hospital, University of Cape Town, Rondebosch, Western Cape, 7925, South Africa; 6 Colorectal Unit, Groote Schuur Hospital, University of Cape Town, Rondebosch, Western Cape, 7925, South Africa; 7 Global Surgery Division, Department of Surgery, Groote Schuur Hospital, University of Cape Town, Rondebosch, Western Cape, 7925, South Africa; 8 Acute Care Surgery Division, Department of Surgery, Groote Schuur Hospital, University of Cape Town, Rondebosch, Western Cape, 7925, South Africa; 9 Faculty of Health and Life Sciences, University of Liverpool, Liverpool, England, L3 5TR, UK

**Keywords:** Patient, Carer, Patient engagement, Infection care, Inclusive

## Abstract

**Background:**

As victims of antimicrobial resistance (AMR) and healthcare recipients, patients and their carers can be engaged in infection prevention and control (IPC) and antimicrobial stewardship (AMS) initiatives to manage AMR. To effectively participate in these initiatives, patients and carers need to understand general terms used in infection care. We explored awareness of commonly used infection-related terms among patients and carers in the surgical out-patient of a tertiary academic hospital.

**Methods:**

Self-administered paper survey distributed among out-patients from August to September 2022. Categorical variables were analysed using Chi squared test. Significance was set as p-value of < 0.05. Content analysis identified terms commonly used by patients when talking about infections.

**Results:**

Overall, 896 out of 1,269 respondents (response rate 70.6%), with a 1:3 male to female ratio were included. Most respondents were patients (75%), with a minimum of high school education (91.2%) and a surgical history (60.3%). *Surgical wound infection* was the most familiar term to participants. While many respondents had not heard of Methicillin-resistant Staphylococcus aureus ( *MRSA*) (92.3%, n=754) or *antimicrobial resistance* (92.8%, n=755), significantly more were aware of the descriptions provided for these terms (13.7% and 33.0%, respectively; p<0.001). Participants considered *antibiotic resistance* to be a condition in which the body rejects, resists, or does not respond to antibiotics.

**Conclusions:**

Findings show dissonance between patients’ awareness of and healthcare workers’ use of infection-care terms, highlighting the need for relatable and accessible terms in infection-care engagement initiatives. More than half of respondents acknowledged that patient engagement responsibility is everyone’s, underscoring the need for contextually fit and relevant communication strategies to advance patient engagement and infection awareness.

## Introduction

Infections are a major threat to safe surgery ^1, 2^. Worldwide, surgical infections contribute to healthcare associated infections (HCAIs) and disease burden, morbidity and mortality ^3^. While HCAIs are preventable, the incidence is on the increase, impacting healthcare costs, with increasing complications given the additional threat of antimicrobial resistance (AMR) ^4, 5^. Low- and middle-income countries (LMICs) are at increased risk of HCAIs such as surgical site infections (SSIs), and report more AMR-related complications and death than their high-income counterparts ^4, 5^. Efforts to contain HCAIs demonstrate the importance of infection prevention and control (IPC) and antimicrobial stewardship (AMS) principles, working in synergy ^2^.

While the surgical pathway is a challenging specialty to engage with respect to stewardship interventions ^6^, the need for greater patient involvement in surgical infection management has been highlighted, especially as a considerable percentage of SSIs occur after hospital discharge ^7^. In South Africa, patients from urban and peri-urban areas present more commonly with post-discharge SSIs than those from rural areas ^8^. Post-discharge SSIs presents an opportunity to engage and include patients in SSI risk management, including AMR risk.

The out-patient department (OPD) of surgical specialties presents an opportunity for improved patient engagement to promote IPC and AMS in surgery. In the out-patient pathway, the patient’s infection-related care and antimicrobial compliance are largely implemented by the patient or the patient’s informal carer once returned to his/her community, and not by a healthcare professional as is the case in-hospital. Management of post-discharge SSIs may be particularly complicated in cases where the infections are resistant to antimicrobials, especially if the patient is unaware of or has incorrect assumptions regarding the basics of infection care and AMR.

Patient and public education has been highlighted as a beneficial investment for the control of AMR infections ^9^, especially when suitably designed for the patient’s context and understanding. Farley *et al.,* ^10^ noted comparably lower knowledge scores on antibiotic resistance in patients attending public healthcare facilities compared to their private healthcare counterparts, and a relationship between knowledge and infection prevention and management attitudes and behaviours. They also recommended further research on required messages for patient engagement interventions ^10^, tailored to the patients’ circumstances and understanding, which may vary from patient to patient.

Research on patients’ understanding of and engagement in infection-related care is limited in surgical settings ^11^, and even more so in the OPD of surgical specialties. Existing research in in-patient settings in South Africa and India has described the important contribution and role of patients and carers in care pathways ^12, 13^. This research explores the patient and carer awareness of infections and infection care terms in the AMR era.

## Methods

### Patient and public involvement

Patients and the public were involved in iterative revision of the study tool, which was continuously revised based on feedback. Patients who want to be informed of the disseminated data had options to provide their contact details or contact the research team through details provided in the participant information leaflet.

### Ethical approval

The study was approved by the Human Research Ethics Committee of the University of Cape Town (Ref: 320/2021) on 29 June 2021, and approval renewed on 21 June 2022. Informed consent was indicated by the participant ticking the relevant box for consent on the paper survey form, which had the study information provided. Participation was voluntary with no risk of prejudice, and participants were made aware of this. Participants who declined participation were noted as such, for calculation of response percentage.

### Study design

A paper-based survey featuring open- and closed-ended questions, was conducted, drawing on previous work of the research team ^14^, with options for free text input where required. Voluntary response sampling was used. The four-part survey included questions on participant demographics, infection care details, knowledge and perceptions of SSI, and knowledge and perceptions of resistant infections. The survey was continuously revised following feedback by members of the public, some of whom were members of the research team. Pilot was iterative until no new ideas for suggestion were made by the study population. Potential participants received the participant information leaflet and revised survey, and participation in the survey was voluntary. The STROBE guidelines ^15, 16^ were adopted for reporting.

### Study participants and setting

Study participants were any adult patients and/or patient carers who visited the hospital at the time of the survey distribution. Surveys were distributed in the waiting areas of the general surgical out-patient, gastrointestinal, cardiac and thoracic, and pharmacy departments at Groote Schuur Hospital, a tertiary academic public healthcare facility in Cape Town, South Africa. With an expected population of 350,000 outpatients annually ^17^, the minimum sample size was calculated as 385, to provide an estimate of the proportion of respondents with awareness of infections and infection care terms among out-patients, with 95% confidence and an alpha level of 0.5 to detect statistical significance, using specific infection care terms as proxy for awareness level.

### Data collection

Data collection took place over a 12-week period from August to October 2022. The surveys were available in IsiXhosa, Afrikaans and English, the three languages predominantly spoken by the population in the area. The survey was piloted with adult patients and/or patient carers who visited the hospital. Members of the research team engaged participants about their understanding of the survey, with several rounds of revisions made following feedback. Patients and/or patient carers who participated in the pilot survey were asked not to participate in the revised survey.

### Data analysis

Data from participants were captured by trained data collectors and capturers and exported to Microsoft Excel (RRID:SCR_016137). Data analysis was performed using IBM SPSS Statistics (Version 27) (RRID:SCR_016479). Respondent characteristics and survey responses were reported using descriptive statistics. The outcomes of interest were awareness of infections (as a surgical risk), including antibiotic resistant infection. They were captured as categorical variables and their frequencies and percentages calculated. Missing data were excluded from the analysis. To assess the association between the response variables (awareness) and the respondent characteristics, Pearson’s Chi-squared test was used and a *p*-value less than 0.05 was considered statistically significant.

In addition to the answer options for various questions, there were also options for free text under the option ‘Other’, to be used by respondents who felt the options included may not reflect their choice. By doing so, we expanded the responses to accommodate any shortcomings that may have influenced the survey questions. Responses provided as free text were analysed using content analysis, by a minimum of two independent reviewers. In cases of disagreement on acceptable codes, such disagreements were resolved by discussion.

## Results

### Demographics

From a total of 1,269 invitations to participate, researchers received 896 (70.6%) survey forms with responses. Close to a third of respondents were male (269, 33.5% – Table 1) ^18^. Most respondents were patients (586, 75.0%) attending various outpatient clinic appointments. Participants aged 30 to 39 and 40 to 49 years old constituted 40% (n=806) of respondents (19.7% and 20.7%, respectively) and 91.2% (733/804) had a minimum of high school education or its equivalent ( Table 1). Of 812 respondents, 60.3% had a surgical history and 29.2% of these (143/490) experienced a pre- or post-surgical infection.

**Table 1.** Self-reported respondent demographics.

Characteristic	n (%)
Sex	n = 803
Male	269 (33.5)
Female	534 (66.5)
Age group	n = 806
Less than 20 years	15 (1.9)
20 – 29 years	114 (14.1)
30 – 39 years	159 (19.7)
40 – 49 years	167 (20.7)
50 – 59 years	150 (18.6)
60 – 69 years	133 (16.5)
Above 70 years	68 (8.4)
Education	n = 804
Primary school (Grade 1 – 7) or equivalent	48 (6.0)
High school (Grade 8 – 12) or equivalent	300 (37.3)
Matric	249 (31.0)
University/College degree	184 (22.9)
Other	23 (2.9)
Care status	n = 781
Patient	586 (75.0)
Carer/Other	195 (25.0)

### Awareness of surgical and related infection terminology

Among respondents, *surgical wound infection* was the term that was more familiar than others, with 45.4% of patients indicating that they had heard of it ( Table 2). Other terms or concepts that respondents were aware of/had heard of were *antibiotic resistance* (307/755, 40.7%), *drug resistance* (293, 38.8%), the provided description of *antimicrobial resistance* (249/754, 33.0%), and the provided description of *antibiotic stewardship* (21.5%). Participants indicated less familiarity with terms such as *superbug*s, a description of Methicillin-resistant Staphylococcus aureus (MRSA), *MRSA* as an acronym, *antimicrobial resistance, antibiotic stewardship*, and *antimicrobial stewardship* ( Table 2). While respondents indicated that they had never heard of some terms used in infection care, a greater number of participants exhibited more awareness of these terms/concepts when they were explained rather than when they were referred to by a term or acronym used in infection care discourse among academic and research stakeholders, *e.g.*, *antibiotic stewardship* and its explanation ( Table 2).

**Table 2.** Percentage of participants aware of infection care terms (n = 754 – 755). MRSA, Methicillin-resistant Staphylococcus aureus.

Have you ever heard of:	Yes	No
MRSA	7.7	92.3
A type of Staph bacteria that is resistant to the antibiotics often used to treat Staph infections	13.7	86.3
Super bugs	16.6	83.4
Drug resistance	38.8	61.2
Antibiotic resistance	40.7	59.3
Antibiotic stewardship	5.3	94.7
The work to measure and improve how doctors give antibiotics to patients and how patients use them	21.5	78.5
Antimicrobial resistance	7.2	92.8
An infection caused by bacteria or fungi or viruses that is resistant to medicines used for treating it	33.0	67.0
Antimicrobial stewardship	2.6	97.4
Surgical wound infection	45.4	54.6

### Source of infection-related information

Concerning awareness and sources of infection-related terms, 43.0% of 865 respondents received such information from healthcare professionals. Other sources of information were traditional and social media (27.7% and 16.5%, respectively), family members (20.2%) and online information sources (17.2%).

### Perceptions of infection care and appropriate antibiotic use

The majority of respondents noted that surgical wound infections can be prevented ( Table 3). Regarding awareness and perceptions surrounding infection care and appropriate antibiotic use, 76% (n = 569) of participants identified bacteria as one type of microbe that is susceptible to antibiotics. Among this population of participants, responses indicated that antibiotics are effective against bacteria only (125, 16.9%), bacteria and viruses (195, 26.2%), most cases of sore throat (186, 25%), and colds and flu (282, 37.8%), and. Most respondents (606, 80%) agreed that responsible antibiotic use helps prevent resistant infections. While 75% of respondents think that misuse of anti-infectives can result in more challenging-to-treat infections, more than half (54.4%) indicated a lack of awareness related to the increasing threat of antibiotic resistance ( Table 3).

**Table 3.** Relationships between selected variables (% response, *n* = 782 – 896).

*Variables*	Surgical wound infections can be prevented (% response)		Antibiotic resistance is increasing all over the world		Misuse/abuse of medicines for infection can make infections more difficult to treat		Antibiotics are effective against bacteria	
Sex	Yes	No/I don’t know	Yes	No/I don’t know	Yes	No/I don’t know	Yes	No/I don’t know
Male	26.2	6.9	16.5	17.5	24.8	8.7	6.5	25.5
Female	53.4	13.6	29.1	36.9	50.0	16.5	10.4	57.6
*p-value*	0.835		0.255		0.723		0.106	
**Age (in years)**								
< 20	1.6	0.4	1.0	1.1	1.3	0.5	0.5	1.4
20 – 29	10.1	3.9	5.5	8.0	9.1	4.7	2.5	11.7
30 – 39	15.8	4.1	9.4	10.8	14.6	5.7	3.4	16.3
40 – 49	16.9	3.8	8.7	11.9	16.7	3.8	3.4	17.2
50 – 59	14.7	3.8	8.7	10.2	13.3	5.5	2.7	16.0
60 – 69	13.1	3.8	8.4	8.6	13.3	3.6	2.9	13.9
> 70	7.0	*0.9*	4.0	3.6	6.2	1.6	1.6	6.4
*p-value*	0.738		0.738		0.062		0.881	
**Education**								
Primary	3.6	1.9	3.2	2.5	3.4	2.2	0.7	4.6
High school	29.3	7.7	16.7	19.9	26.4	10.7	4.1	32.7
Matric	24.3	6.9	12.2	18.9	23.4	7.5	5.3	26.5
University/College	20.1	3.4	12.7	10.9	19.5	3.8	6.5	16.8
Other	2.0	0.7	1.1	1.8	2.0	1.1	0.4	2.3
*p-value*	0.054		0.030		0.005		0.001	
**Care status**								
Patient	58.7	16.5	32.8	42.2	55.6	19.0	12.9	61.7
Carer/Other	20.9	3.9	13.3	11.7	19.2	6.3	4.2	21.2
*p-value*	*0.069*		*0.030*		*0.806*		*0.861*	

### Statistical relationships between study variables

There were no statistically significant differences in participants’ responses to the questions on prevention of surgical wound infections, increasing global threat of antibiotic resistance, misuse or abuse of anti-infectives as a risk for AMR, and the use of antibiotics for bacterial infections across demographic variables such as sex and age (p-values > 0.05) ( Table 3). Differences in responses were, however, seen in responses to some questions according to the participant’s education and/or care status, with 38.8% and 59.2% of high school attendants/those with high school matric unaware of the increasing threat of ABR or the efficacy of antibiotics against bacteria, respectively. Participants who identified as *Carer/Other* were more aware of the increasing threat of antibiotic resistance than participants who identified as patients ( Table 3).

Significant differences were highlighted between respondents who indicated awareness of the term, *MRSA*, versus those who indicated awareness of the explained concept, X ^2^(1, N = 754) = 91.804, p < 0.001). This was also noted for the terms, *antibiotic resistance* and *antimicrobial resistance* versus the explanation – *an infection caused by bacteria or fungi or viruses that is resistant to medicines used for treating it*, X ^2^(1 *, n* = 755) = 114.000 and X ^2^(1, *n* = 755) = 40.520, respectively.

### Perceptions of SSIs, ABR and options for patient and public engagement

***Respondent perceptions of SSIs*.** Content analysis of free text responses (provided mostly when respondents chose the option, *Other*, and included details of this *Other*) highlighted respondents’ views on surgical site infection, antibiotic resistance, and patient and public engagement options. Between 300 and 350 respondents provided no response on the questions about knowledge/understanding of SSI, ABR, and options for patient engagement. Among 569 respondents, the terms *surgical wound*, *surgery*/ *operation* and *infection* largely featured in descriptions of SSIs, with 15.8% describing this as an infection of a surgical wound. SSIs were seen to occur in cases where the surgical wound is not cared for/appropriately treated (11.4%), hygiene practices are irregular/lacking (8.6%) and were linked to bacteria/microbes (7.6%).

***Respondent perceptions of ABR*.** Among respondents who provided a description for ABR ( *n* = 518), antibiotic resistance was seen more as a condition in which the *body* plays a role to *reject, resist* or not *respond* to an antibiotic, rather than bacteria acquiring resistance. Some participants’ responses showed that antibiotics were *not working* in cases of resistance, with some seeing the condition to be incurable/untreatable. Six respondents indicated that antibiotic resistance occurs in cases where bacteria overcome drugs/antibiotics meant to destroy or kill them.

***Respondents’ preference for patient and public engagement*.** Education (67/529) and improved awareness (57/529) were noted as avenues for patient and carer engagement in infection care. Several platforms were advocated for this, with social media (195/552) suggested by a higher number of participants compared to other platforms. Whilst 52.1% (451/865) considered promoting responsible antibiotic use to be everyone’s responsibility, 25% (301) considered this to be the responsibility of those who work in hospitals/healthcare workers and government (216).

## Discussion

The study aimed to explore the awareness and perceptions of patients and patient carers around specific commonly encountered/used terms and concepts used in infection-related care. Of all the terms assessed, respondents were most familiar with the term *surgical wound infection*. Previous studies reported low levels of SSI awareness ^19^, and deficiencies in discharge planning, including education on surgical wound care, as contributing to post-discharge concerns and infection-related complications ^7, 19^. Studies that explored patient experiences of SSIs in three UK hospitals reported patients’ lack of SSI awareness, and the role(s) that healthcare staff could have played during a patient’s hospital stay as important in helping patient understanding and perceptions ^20^.

Respondents in our study were less aware of infection care terms such as antibiotic resistance, MRSA, and stewardship, though significantly more were aware of the concepts inferred by these terms. The difference highlights an important opportunity to increase understanding and engender the correct perceptions by concentrating on explaining concepts. Of note, a study by Tanner *et al.*, ^20^ highlighted patients’ and sometimes, healthcare workers’ perceptions of MRSA as a more dangerous infection than *other* infections, including SSIs, which were seen as random occurrences *versus* MRSA, which was seen as an avoidable infection caused by facility-level negligence. Our findings of low levels of understanding relating to the difference between bacteria becoming resistant to antibiotics rather than humans becoming resistant is in keeping with previous studies ^21, 22^, drawing attention to concepts that require clarification.

The use of language relating to AMR in public engagement and communication campaigns fails to make infection awareness relatable ^23, 24^. Recommendations have been made for simpler language, which focuses on illness and its implications, to improve public engagement with the threat of AMR ^23, 24^. This also underscores the need for healthcare workers to be aware of the need to better communicate general infection-related terms, including AMR-related information. Infection-related information as explored in this study, was generally provided by healthcare workers. As reported in a previous study ^25^, other important sources of infection-related information for patients and carers were online and traditional media, which provide opportunities for wider stakeholder involvement.

Inappropriate antibiotic prescribing behaviours and patient/carer expectations of receiving antibiotics for viral infections such as colds, flu, and diarrhoea, are well described, and have been recognized as drivers of antibiotic resistance ^26– 28^. Stewardship promoting initiatives to address these drivers have been suggested for engagement areas. For example, antibiotic expectation behaviour in patient populations reduced when information about antibiotic efficacy was provided and no difference in expectations when information on viral aetiology of the illness was provided ^27^. This illustrates that a positive outlook to antibiotic stewardship can be elicited by the timely provision of relatable information, arguably more effective when relatable language that speaks to the patients’ needs ( *e.g.*, *drug efficacy* versus *viral aetiology*) is used.

While most survey respondents are aware that responsible antibiotic use can prevent AMR, they did not necessarily see use for viral infections as inappropriate. Overall, respondents shared that everyone, including patients and the public, should be involved in spreading messages on appropriate antibiotic use. Insight from our research indicates that healthcare workers provide health-related information, including infection care information, to patients. Patients in another study have, however, reported confusion and unclear communication about illnesses, which may be treated with antibiotics, highlighting gaps such as the importance of easy-to-understand educational materials to facilitate healthcare worker-patient engagement and communication in antibiotic stewardship initiatives ^25^. Suitable adaptation of communication strategies in the context of patient consultations is also needed to address inappropriate antibiotic prescribing and expectations ^29^. This also underscores the need for additional work in patient engagement for responsible antibiotic use.

Respondents, while noting that misuse of anti-infectives can result in more challenging-to-treat (resistant) infections, were largely unaware of the increasing threat of antibiotic resistance. This further supports stewardship roles for patients/the public ^30^. Drivers of antibiotic prescribing, expectation and use may sometimes be more related to social, behavioural and environmental factors rather than biomedical factors ^28, 31^, and so, effective intervention strategies need to consider such wider factors.

### Strengths and limitations

Our study highlights dissonance between patients’ awareness of terms commonly used by healthcare workers and researchers in infection care discourse. We acknowledge limitations that need to be considered in the interpretation.

As a cross-sectional study, it cannot determine temporal relationships. Relevance may also change over time. The distribution of the survey in paper form may have limited participation by those who may have preferred to participate online. In addition, sampling was by voluntary response rather than random or stratified. Respondents are therefore not representative of patients or carers at the study site, and so, findings cannot be generalized.

Nevertheless, this study fills a gap in knowledge and underscores the need for relatable terms to be used in patient and carer engagement for infection care. It also highlights the pivotal role of language in effective communication. Future research can explore ways to employ the tools and platforms, identified by participants, while incorporating accessible and relatable information, for development of locally relevant patient engagement framework.

## Conclusions

Our study shows the disparity between patients’ understanding of infection care concepts and the terms we use to reference them during infection care. We need to use more inclusive language and expressions in patient and wider stakeholder engagement initiatives, if our patients, patient carers, and the public are to become engaged in infection care. Given respondents’ reliance on healthcare workers for information, an opportunity exists for enhancing staff awareness concerning the pivotal role that language plays in effectively communicating and educating patients and the wider population about SSIs, their risks and management, as well as antibiotic use, resistance, and stewardship. This is vital as initiatives to evaluate patient and wider public understanding of infection care using unfamiliar and unrelatable language may present inaccurate reflection of patient awareness, in addition to highlighting researcher’s inexperience of contextual factors. Furthermore, the findings suggest that initiatives designed to involve patients and the public in infection care should strategize towards using more traditional as well as social media platforms, given their potential for raising awareness.

According to this study, the scope of responsibility for patient engagement not only falls on healthcare workers, but also on patients as well as their carers/families and the larger population, given their place in the healthcare pathway and their contribution to antibiotic use. Consequently, this research highlights the need for contextually fit and inclusive communication strategies within the domain of infection care, to advance patient engagement for improved health outcomes.

## Data Availability

Figshare: Awareness of infection care terms among outpatients and carers in a public health facility: a cross-sectional survey. https://doi.org/10.6084/m9.figshare.24216777 ^18^. Figshare: STROBE checklist for ‘Awareness of infection care terms among outpatients and carers in a public health facility: a cross-sectional survey’. https://doi.org/10.6084/m9.figshare.24412714 ^16^. Data are available under the terms of the Creative Commons Attribution 4.0 International license (CC-BY 4.0).
