# Supplementary material for: Awareness of infection care terms among outpatients and carers in a public health facility: a cross-sectional survey
Source: Wellcome Open Res. 2024 Oct 9;8:574. Originally published 2023 Dec 14. [Version 2] doi: 10.12688/wellcomeopenres.20162.2 (PMC11523555; doi:10.12688/wellcomeopenres.20162.2)
Supplement: Peer review history

# Reviewer report: Reviewer response for version 2

**DOI:** 10.21956/wellcomeopenres.25528.r104476

**Published:** 2024-11-01

**Authors:** Wuraola Akande-Sholabi

**Affiliations:** 1 Department of Clinical Pharmacy and Pharmacy Administration, Faculty of Pharmacy, University of Ibadan, Ibadan, Oyo, Nigeria

Thank you for giving me the opportunity to review this manuscript which addresses the awareness of infection care terms among outpatients and carers in public health facility: a cross sectional survey.

 The manuscript is well written and the research was carried appropriately and can be easily reproduced. 

 Comment

 I will like the authors to clarify why in the discussion section, they have included this statement "Long-term solutions to improving infection care, including SSI and ABR awareness, may include integration of AMR and SSI education in health care programmes and academic curricula".

 I feel the statement is out of place and does not reflect any findings from their research results and should not be included. The study has not showed that healthcare professionals are not aware of SSI, ABR and AMR.

Is the work clearly and accurately presented and does it cite the current literature?

Yes

If applicable, is the statistical analysis and its interpretation appropriate?

Yes

Are all the source data underlying the results available to ensure full reproducibility?

Yes

Is the study design appropriate and is the work technically sound?

Yes

Are the conclusions drawn adequately supported by the results?

Yes

Are sufficient details of methods and analysis provided to allow replication by others?

Yes

Reviewer Expertise:

Global Health, Antimicrobial stewardship, antimicrobial resistance, inappropriate use of medications with a focus on the elderly.

I confirm that I have read this submission and believe that I have an appropriate level of expertise to confirm that it is of an acceptable scientific standard.

# Reviewer report: Reviewer response for version 2

**DOI:** 10.21956/wellcomeopenres.25528.r104188

**Published:** 2024-10-28

**Authors:** Syed Hassan Ahmed

**Affiliations:** 1 Internal Medicine, Dr Ruth KM Pfau Civil Hospital Karachi, Karachi, Sindh, Pakistan

The authors have adequately addressed the mentioned comments.

Is the work clearly and accurately presented and does it cite the current literature?

Yes

If applicable, is the statistical analysis and its interpretation appropriate?

Yes

Are all the source data underlying the results available to ensure full reproducibility?

Yes

Is the study design appropriate and is the work technically sound?

Yes

Are the conclusions drawn adequately supported by the results?

Yes

Are sufficient details of methods and analysis provided to allow replication by others?

Partly

Reviewer Expertise:

Internal Medicine

I confirm that I have read this submission and believe that I have an appropriate level of expertise to confirm that it is of an acceptable scientific standard.

# Reviewer report: Reviewer response for version 1

**DOI:** 10.21956/wellcomeopenres.22325.r74220

**Published:** 2024-04-25

**Authors:** Md. Saiful Islam

**Affiliations:** 1 School of Population Health, University of New South Wales, Sydney, New South Wales, Australia

This manuscript is exceptionally well-written, effectively communicating both the results and the discussion. However, the authors could enhance the discussion by providing further insight into the importance of understanding infection care terms, especially the highly technical terminology.

 In the methods section, it is crucial for the authors to clearly define who the carers are. This clarification will help readers better understand the study's participants and the context of their involvement.

 Furthermore, the manuscript would benefit from elaborating more on how these findings can contribute to policy development. Specifically, the authors should outline short-term recommendations for immediate implementation, as well as long-term recommendations for sustained impact. Additionally, discussing the best strategies for engaging patients and their carers in infection care initiatives would be valuable.

 By addressing these points, the manuscript can provide a more comprehensive understanding of the implications of the study findings and their potential impact on both policy and practice in infection care.

Is the work clearly and accurately presented and does it cite the current literature?

Yes

If applicable, is the statistical analysis and its interpretation appropriate?

Yes

Are all the source data underlying the results available to ensure full reproducibility?

Partly

Is the study design appropriate and is the work technically sound?

Yes

Are the conclusions drawn adequately supported by the results?

Yes

Are sufficient details of methods and analysis provided to allow replication by others?

Yes

Reviewer Expertise:

Public health surveillance, outbreak investigation, infection prevention and control, antimicrobial resistance and social science intelligence in epidemic preparedness and response

I confirm that I have read this submission and believe that I have an appropriate level of expertise to confirm that it is of an acceptable scientific standard.

# Response

**Published:** 2024-09-28

**Authors:** Oluchi Mbamalu

**Affiliations:** Medicine, University of Cape Town, Observatory, Western Cape, South Africa

**Responses to Reviewer 2 **   Dear Dr Md Saiful Islam, We would like to thank you for reviewing our  manuscript and for the opportunity to respond to your comments. Please find below responses to the comments made.

**Reviewer's Comment: **This manuscript is exceptionally well-written, effectively communicating both the results and the discussion. However, the authors could enhance the discussion by providing further insight into the importance of understanding infection care terms, especially the highly technical terminology.

**Response: **The authors thank the reviewer for this positive comment. In two paragraphs at the end of the *Discussion *section, we have further elaborated on the importance of patients' understanding of infection care terminology. We further highlighted the significance of patients' increased understanding of highly technical SSI and other infection care terminology, and how this can influence efforts towards addressing AMR.  

**Reviewer's Comment: **In the methods section, it is crucial for the authors to clearly define who the carers are. This clarification will help readers better understand the study's participants and the context of their involvement.

**Response: **In this study, *carer* refers to anyone who provided support and assistance to the patient. This could be an employed help or family member such as parent, guardian, sibling or spouse. We have clarified this in the *Methods *section; please see details under *Study participants and setting*.

**Reviewer's Comment: **Furthermore, the manuscript would benefit from elaborating more on how these findings can contribute to policy development. Specifically, the authors should outline short-term recommendations for immediate implementation, as well as long-term recommendations for sustained impact. Additionally, discussing the best strategies for engaging patients and their carers in infection care initiatives would be valuable.

 By addressing these points, the manuscript can provide a more comprehensive understanding of the implications of the study findings and their potential impact on both policy and practice in infection care. 

**Response: **In the last two paragraphs of the *Discussion *section, we have included some contextually relevant suggestions on how these findings can inform local policy development and practice. We have also included long term recommendations for sustained impact, as well as suggestions on how patient and carer engagement in infection care initiatives can be advanced.   

 Thank you again for your review and insights which have improved this manuscript.    Yours sincerely,  O Mbamalu & E R Useh *On behalf of the co-authors*.

# Reviewer report: Reviewer response for version 1

**DOI:** 10.21956/wellcomeopenres.22325.r75730

**Published:** 2024-03-14

**Authors:** Syed Hassan Ahmed

**Affiliations:** 1 Internal Medicine, Dr Ruth KM Pfau Civil Hospital Karachi, Karachi, Sindh, Pakistan

The article highlights an important aspect of surgical care, assessing awareness of commonly used infection-related terms. The study indicates a dire need to promote understanding of commonly used infection care terms among patients and their caregivers to improve their understanding of potential surgical outcomes and complications. However, here are a few suggestions that authors can incorporate: 

1. Was a standardised questionnaire used? If yes, kindly provide details about it. In case if the researchers self-designed the questionnaire, was it validated? Similarly, were the translations validated?
2. Was there any particular exclusion criteria?
3. Under “Perceptions of infection care and appropriate antibiotic use”, the following sentence is incomplete: “Among this population of participants, responses indicated that antibiotics are effective against bacteria only (125, 16.9%), bacteria and viruses (195, 26.2%), most cases of sore throat (186, 25%), and colds and flu (282, 37.8%), and.” Either remove adjust the error or complete the sentence.
4. Any plausible reason why were carers more aware than participants?
5. It will be amazing if the authors can draw some statistical comparisons between their findings, relative to studies conducted in other high and low-income countries.
6. Lastly, it will be a good addition if authors can add in a few words about which online platforms can be employed to raise awareness, or any other strategy. 

Is the work clearly and accurately presented and does it cite the current literature?

Yes

If applicable, is the statistical analysis and its interpretation appropriate?

Yes

Are all the source data underlying the results available to ensure full reproducibility?

Yes

Is the study design appropriate and is the work technically sound?

Yes

Are the conclusions drawn adequately supported by the results?

Yes

Are sufficient details of methods and analysis provided to allow replication by others?

Partly

Reviewer Expertise:

Psychiatry; mental health

I confirm that I have read this submission and believe that I have an appropriate level of expertise to confirm that it is of an acceptable scientific standard, however I have significant reservations, as outlined above.

# Response

**Published:** 2024-09-28

**Authors:** Oluchi Mbamalu

**Affiliations:** Medicine, University of Cape Town, Observatory, Western Cape, South Africa

**Responses to Reviewer 1 ** 10 April 2024 Dear Dr Ahmed, Thank you for reviewing our manuscript and for the opportunity to respond to your comments. Please find below a point-by-point response to the comments made.

1. Was a standardised questionnaire used? If yes, kindly provide details about it. In case if the researchers self-designed the questionnaire, was it validated? Similarly, were the translations validated?

**Response:       **A standardised questionnaire was used for the survey. It was designed by the research team and revised following pilots in the study population, until no further revisions were recommended. The questionnaire was administered to participants who were asked the same questions in identical formats, with response options provided in the same format. Translations of the questionnaire were included in the pilot study, and revised according to feedback received.  We have included this under *Study participants and setting *(page 3 of 11).  

1. Was there any particular exclusion criteria?

**Response:       **The survey was administered to out-patients and carers; patients admitted to the hospital were not included as participants in the survey.                         We have included this under *Study participants and setting *(page 3 of 11).  

1. Under “Perceptions of infection care and appropriate antibiotic use”, the following sentence is incomplete: “Among this population of participants, responses indicated that antibiotics are effective against bacteria only (125, 16.9%), bacteria and viruses (195, 26.2%), most cases of sore throat (186, 25%), and colds and flu (282, 37.8%), and.” Either remove adjust the error or complete the sentence.

**Response:       **We thank the reviewer for this; we have revised accordingly.  

1. Any plausible reason why were carers more aware than participants?

**Response:      **It may be that carers have been exposed to infectious conditions when caring for (an)other patient(s) whereas patients may not have had such exposure; however, this is not certain. We are exploring this finding in further engagement work with patients and carers.  

1. It will be amazing if the authors can draw some statistical comparisons between their findings, relative to studies conducted in other high and low-income countries.        

**Response: **The authors thank the reviewer for this wonderful perspective; we have included statistical data from other studies that reported on the topics discussed in this manuscript – please see edits in the *Discussion *section.   

1. Lastly, it will be a good addition if authors can add in a few words about which online platforms can be employed to raise awareness, or any other strategy. 

**Response:       **Many participants mentioned social media/digital media without being more specific. Among those who were specific, Facebook and WhatsApp were commonly mentioned. ****We have included this in the section, *Source of infection-related information* in page 4 of 11.   Yours sincerely,  Oluchi Mbamalu *On behalf of the co-authors.*
